# Whole-genome sequence data and analysis of a *Staphylococcus aureus* strain SJTUF_J27 isolated from seaweed

**DOI:** 10.1016/j.dib.2018.08.084

**Published:** 2018-08-30

**Authors:** Yanping Xie, Yiping He, Sandeep Ghatak, Peter Irwin, Xianghe Yan, Terence P. Strobaugh, Jr., Andrew Gehring

**Affiliations:** a Molecular Characterization of Foodborne Pathogens Research Unit, Eastern Regional Research Center, Agricultural Research Service (ARS), United States Department of Agriculture (USDA), 600 East Mermaid Lane, Wyndmoor, PA 19038, USA; b Division of Animal Health, ICAR Research Complex for NEH Region, Umiam, Meghalaya 793103, India; c Environmental Microbial and Food Safety Laboratory, Beltsville Agricultural Research Center, ARS, USDA, 10300 Baltimore Avenue, Beltsville, MD 20705, USA

**Keywords:** *Staphylococcus aureus*, Genome assembly, Whole genome sequencing (WGS), Virulence factor

## Abstract

The complete genome sequence data of *S. aureus* SJTUF_J27 isolated from seaweed in China is reported here. The size of the genome is 2.8 Mbp with 32.9% G + C content, consisting of 2614 coding sequences and 77 RNAs. A number of virulence factors, including antimicrobial resistance genes (fluoroquinolone, beta-lactams, fosfomycin, mupirocin, trimethoprim, and aminocoumarin) and the *egc* enterotoxin cluster, were found in the genome. In addition, the genes encoding metal-binding proteins and associated heavy metal resistance were identified. Phylogenetic data analysis, based upon genome-wide single nucleotide polymorphisms (SNPs), and comparative genomic evaluation with BLAST Ring Image Generator (BRIG) were performed for SJTUF_J27 and four *S. aureus* strains isolated from food. The completed genome data was deposited in NCBI׳s GenBank under the accession number CP019117, https://www.ncbi.nlm.nih.gov/nuccore/CP019117.

## Specifications Table

**Table:** 

Subject area	Biology
More specific subject area	Microbial genomics
Type of data	Completed genome sequence in FASTA format, figures
How data was acquired	Illumina Miseq sequencing platform
Data format	Analyzed
Experimental factors	*Staphylococcus aureus* SJTUF_J27 isolated from seaweed
Experimental features	Whole genome sequencing, *de novo* assembly, and annotation
Data source location	Shanghai, China (Latitude 31.23 N and Longitude 121.47 E)
Data accessibility	Data is with this article and available online at https://www.ncbi.nlm.nih.gov/nuccore/CP019117

## Value of the data

• The complete genome sequence of *S. aureus* SJTUF_J27, which was isolated from Chinese seaweed, provides a genetic basis for understanding the epidemiology of food-associated staphylococci.
• The sequence data will be useful for comparative genomic study of *S. aureus.*
• Analyses of virulence and antibiotic resistance genes can be used to predict the probability of the organism being a multidrug resistance pathogen.
• The genome-wide SNP analysis generated a high-resolution phylogenetic tree of *S. aureus* food isolates, which is a useful tool for accurately discriminating closely related species.

## 1 Data

**Fig. 1:**
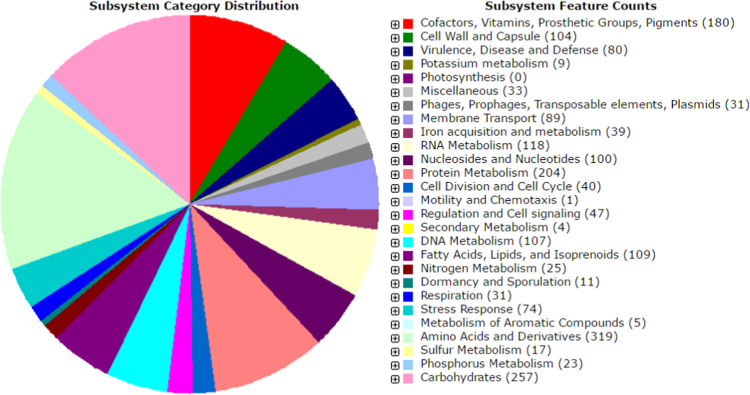
Subsystem categories and distribution of the *S. aureus* SJTUF_J27 genome annotated by RAST.

With an average of 331-fold sequencing coverage, a genome size of 2,804,759 bp constituting 32.9% of G + C content was generated. RAST annotation of the genome revealed a total of 399 subsystems, 2614 coding sequences (80 of them related to virulence, disease and defense), and 77 RNAs (Fig. 1). PathogenFinder showed the probability of this strain being a human pathogen was 98%.

Analysis of the genomic data showed the organism contains several antimicrobial resistance genes, including fluoroquinolone resistance-determining region of *gyrA*, *gyrB*, *parC* and *parE*, teicoplanin-associated operon of *tcaR*-*tcaA*-*tcaB*, beta-lactamase genes, and fosfomycin resistance gene *fosB*. Comprehensive Antibiotic Resistance Database (CARD) identified mupirocin resistance mediated by *ileS*, trimethoprim resistance mediated by *dfrC*, and aminocoumarin resistance mediated by *alaS*. The strain harbors heavy metal resistance genes and the enterotoxin gene cluster (*egc*) but lacks staphylococcal pathogenicity islands (SaPI).

**Fig. 2:**
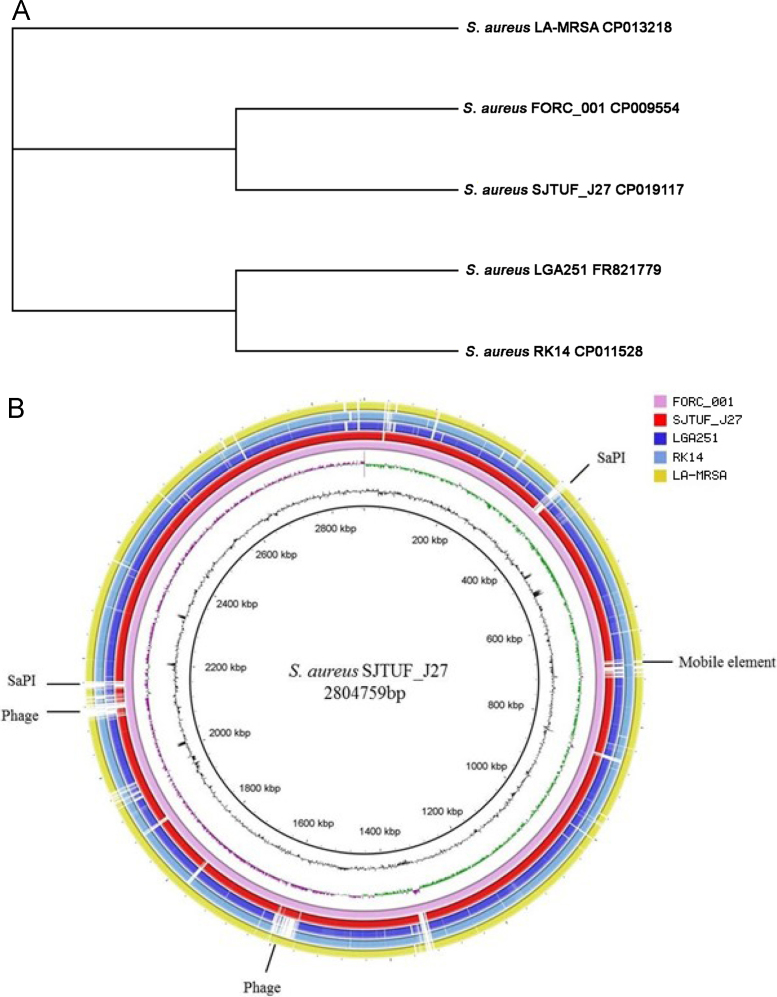
A. Phylogenetic tree of *S. aureus* strains based on whole genome single-nucleotide polymorphisms. B. BRIG ring comparisons of the *S. aureus* strains SJTUF_J27, FORC_001, LGA251, RK14, and LA-MRSA. The main divergent regions are labeled with SaPI, phage and mobile element. The innermost rings represent the GC content (black) and GC skew (purple/green) of FORC_001.

Genome-wide single nucleotide polymorphism analysis revealed the phylogenetic relationships of the strain to four food isolates (*S. aureus* FORC_001, LGA251, RK14 and LA-MRSA with accession numbers CP009554, FR821779, CP011528 and CP013218, respectively) (Fig. 2A). BRIG analysis showed the differences between these strains mainly in the mobile genetic elements of phage and SaPI (Fig. 2B). Furthermore, MLST (http://www.mlst.net) showed that SJTUF_J27 belongs to sequence type (ST)433, and all these food isolates belong to different STs (FORC_001 to ST30, LGA251 to ST425, RKI4 to ST8, and LA-MRSA to ST398).

## 2 Experimental design, materials and methods

*S. aureus* SJTUF_J27 was isolated from seaweed in China. Identification of the strain was carried out using the API Staph-Ident system (bioMerieux, Shanghai, China). The result was confirmed by 16s rRNA sequencing [1]. For genome sequencing, DNA was extracted using DNeasy Blood & Tissue Kit (Qiagen, Valencia, CA), quantified by a Qubit 2.0 fluorometer (Thermo Fisher Scientific, Waltham, MA), and then subjected to library construction using the Nextera XT sample preparation kit (Illumina, San Diego, CA). Next-generation sequencing was performed in Illumina Miseq platform with 2 × 300 paired-end sequencing chemistry. A total of 1,547,292 raw sequence reads were automatically generated, trimmed for quality, and then *de novo* assembled using the CLC genomics workbench v 9.5 and SPAdes 3.9. The assembled genome was validated by Sanger sequencing and mapping reads back to the assembly. The complete genome sequence of *S. aureus* SJTUF_J27 was deposited to NCBI under the accession number CP019117. Annotation of the genome was performed using the Rapid Annotation Subsystem Technology (RAST) sever (http://rast.nmpdr.org/) [2]. Pathogenicity and antibiotic resistance were predicted using PathogenFinder (https://cge.cbs.dtu.dk/services/PathogenFinder/) and Comprehensive Antibiotic Resistance Database (CARD) (https://card.mcmaster.ca/) [3], respectively. Single nucleotide polymorphism (https://cge.cbs.dtu.dk/services/CSIPhylogeny/) [4], BLAST Ring Image Generator (BRIG) (https://sourceforge.net/projects/brig/) [5], and MLST (http://www.mlst.net) were used for comparative analyses of the *S. aureus* food isolates.
